# Feasibility of stereotactic radiotherapy with pembrolizumab in patients with deficient mismatch repair/microsatellite unstable metastatic colorectal cancer

**DOI:** 10.1016/j.esmogo.2024.100069

**Published:** 2024-06-14

**Authors:** A. Gandini, V. Martelli, L. Belgioia, S. Puglisi, M. Cremante, V. Murianni, A. Damassi, C. Pirrone, F. Catalano, M. Grassi, L. Trevisan, S. Vagge, V. Andretta, S. Mammoliti, D. Comandini, G. Fornarini, A. Pessino, A. Pastorino, S. Sciallero, A. Puccini, A.F. Sobrero

**Affiliations:** 1 Department of Internal Medicine and Medical Specialties (Di.M.I.), University of Genoa, Genoa, Italy; 2 Medical Oncology Unit 1, IRCCS Ospedale Policlinico San Martino, Genoa, Italy; 3 Health Science Department (DISSAL), University of Genoa, Genoa, Italy; 4 Radiation Oncology Department, IRCCS Ospedale Policlinico San Martino, Genoa, Italy; 5 Unit of Hereditary Cancer, IRCCS Ospedale Policlinico San Martino, Genoa, Italy; 6 Radiotherapy Department, E.O. Ospedali Galliera, Genoa, Italy

**Keywords:** immunotherapy, radiotherapy, colorectal cancer, immunomodulation, abscopal effect, combined modality therapy

## Abstract

**Background:**

Patients with metastatic colorectal cancer (mCRC) carrying a deficit in the mismatch repair system/microsatellite instability (dMMR/MSI) show great responses to immune checkpoint inhibitors. However, 30% of patients with dMMR/MSI are primarily immunoresistant, and another 30% develop secondary resistance. Thus several combinations such as anti-programmed cell death protein 1 (anti-PD-1) and anti-cytotoxic T-lymphocyte-associated protein 4 (anti-CTLA-4) are being pursued. The combination of radiotherapy and immunotherapy is another avenue of research that can increase the release of neoantigens resulting in the abscopal effect. This phenomenon has demonstrated promising potential activity in colon cancer preclinical studies; nevertheless, clinical results are limited to just a few case series.

**Patients and methods:**

We conducted a prospective interventional single-institution study to assess the feasibility, safety, and disease control rate of the combination of pembrolizumab and stereotactic ablative radiotherapy (SABR) in a cohort of 14 consecutive patients with dMMR/MSI mCRC.

**Results:**

Among the 14 patients enrolled, 11 received SABR in combination with pembrolizumab as the first to the fourth line. The disease control rate was 50% in the intention-to-treat population, with six patients still maintaining the response after >15 months. Any-grade treatment-related adverse events occurred in 50% of patients, with grade 3 (G3) events in three patients; no treatment-related death occurred.

**Conclusions:**

Our findings convey no signal of enhanced systemic efficacy compared with historical data on pembrolizumab alone even if the local control rate is high. To our knowledge, this represents the largest study conducted in this population; further studies could extend the knowledge on the toxicity profile of this combination.

## Highlights

• ORR of pembrolizumab + stereotactic radiotherapy in dMMR/MSI mCRC was 50%.
• The activity level reported is in the range of that expected with anti-PD-1 alone.
• No signal of enhanced systemic efficacy was seen, despite high local control.
• The toxicity rate reported raises an issue about the tolerability of this combination.

## Introduction

Metastatic colorectal cancer (mCRC) treatment is guided by few actionable biomarkers and, among these, microsatellite instability (MSI)/DNA mismatch repair deficiency (dMMR) has shown outstanding results as a predictive biomarker of large benefits from immune checkpoint inhibitors (ICIs). Because of the accumulation of high levels of single-base mismatches, short insertions, or deletions in repetitive DNA tracts,^1^ an increased number of mutation-associated neoantigens are released, creating an immunogenic tumor microenvironment that underlies the response to immunotherapy.^2^ In mCRC this effect was observed in both first^3^^,^^4^ and later lines^5^ of therapy no matter how advanced the conditions of these patients.

However, not all patients with dMMR/MSI mCRC achieve response and long-term benefit from immunotherapy. In general, ∼30% of patients do not respond to pembrolizumab (KEYNOTE-177),^3^ and another 30%-40% will develop secondary resistance and progress during ICI therapy in the first line. Thus combination strategies are under investigation to overcome the mechanisms of resistance.^6^ In the last years, some evidence has shown that radiotherapy can enhance both immune response and clinical outcomes in several solid tumors^7^, ^8^, ^9^, ^10^, ^11^, ^12^ with rates of toxicity similar to that of radiotherapy or immunotherapy alone.^11^ The rationale for the use of this combination is the abscopal effect, which refers to the phenomenon of tumor regression at a distant site from the one irradiated^13^ through the systemic release of antigens from tumor tissue,^14^ which promotes an immune response against tumor tissue both locally and at distance.^15^^,^^16^

The combination of immunotherapy and radiotherapy has shown promising results in some malignancies, such as non-small-cell lung cancer^17^^,^^18^ and melanoma,^19^ while efficacy in CRC remains widely unknown. In particular, preclinical studies on breast and colon carcinoma cells and mice suggested that this combination strategy could potentially turn immunotherapy-resistant tumors into immunologically responsive ones,^20^^,^^21^ but data on its clinical application are missing.

Because of these reasons, we conducted a prospective interventional single-institution study to assess the feasibility of the combination of pembrolizumab and stereotactic ablative radiotherapy (SABR) in a series of 14 consecutive patients with dMMR/MSI mCRC.

## Patients and methods

### Study population

Eligible patients had:

• histologically confirmed metastatic MSI or dMMR CRC with measurable disease according to RECIST version 1.1, as confirmed by radiologic assessment;
• Eastern Cooperative Oncology Group (ECOG) performance status ≤2;
• no disease requiring concomitant use of corticosteroid therapy exceeding the equivalent of 10 mg/day prednisone;
• no disease contraindicating the use of anti-programmed cell death protein 1 (anti-PD-1) therapy; and
• no previous therapy with anti-PD-1/anti-programmed death-ligand 1 (anti-PD-L1) agents.

### Study design and treatment

This monocentric interventional prospective study was conducted at IRCCS Ospedale Policlinico San Martino in Genoa, Italy. Patients who met the inclusion criteria received pembrolizumab 200 mg intravenously every 21 days until progression, according to immune RECIST (iRECIST), treatment intolerance, or up to 35 cycles of total treatment. Dose interruptions were permitted for treatment-related adverse events (TRAEs), but dose modifications were not allowed. Patients with radiological disease progression who experienced clinical benefit could be treated beyond progression in accordance with the physician’s decision.

In this pilot study, SABR was carried out according to the following feasibility criteria:

• one or more targetable metastases, independent of the disease site;
• timing administration concurrent with the second or third cycle of pembrolizumab;
• two sessions with mono-weekly frequency; and
• 15-Gray fraction (considered as ablative, to achieve local control of the disease) followed by a 5-Gray fraction (considered as the fraction able to enhance the immunogenic effect).

Peritoneal sites were not irradiated due to variability in the target location.

All patients, before SABR, underwent a computed tomography scan simulation in the treatment position with the aid of a thermoplastic mask or a four-dimensional computed tomography simulation for lung lesions. Image fusion with diagnostic examinations was applied to optimize target volume delineation and different organs at risk were contoured depending on the site of the disease and of the healthy nearby structures. SABR was delivered using intensity-modulated radiotherapy or volumetric-modulated arc therapy. Daily image guidance was constantly applied to verify patient positioning and set up accuracy before each fraction. The dose prescription required that 95% of the planning target volume was covered by a minimum of 95% of the prescribed dose.

The primary endpoint was feasibility, defined as compliance, adherence to the administration timing, and safety in a real-world setting. The secondary endpoints were response rate, according to the iRECIST criteria, and disease control, defined as not progression [i.e. complete response, partial response, or stable disease].

### Assessments

Mismatch repair status was determined on CRC tissue by immunohistochemical analysis of the proteins coded by the mismatch repair genes *MLH1, MSH2, MSH6*, and *PMS2.* The diagnosis of dMMR tumor occurred in case of loss of expression of MMR proteins. The MSI status was, instead, determined locally by PCR-based analysis. The *BRAF* status was evaluated in immunohistochemical analysis or through mass spectrometry. The *RAS* status was evaluated in PCR.

Tumor response was assessed according to iRECIST criteria (version 1.1) by imaging with a computed tomography scan of the chest–abdomen–pelvis, magnetic resonance imaging, or positron emission tomography; specific tumor markers and/or clinical examination were carried out at baseline and every 2 months or whenever progression was clinically suspected.

Adverse events were reported throughout the study and were graded according to the National Cancer Institute Common Terminology Criteria for Adverse Events (CTCAE), version 4.0.

## Results

### Patient characteristics

**Figure 1:**
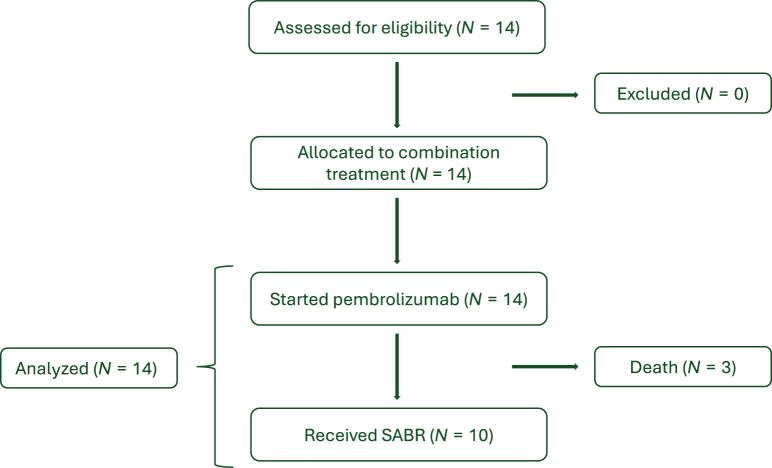
**Study flowchart**. SABR, stereotactic ablative radiotherapy.

Between November 2019 and April 2021, 14 unselected patients with dMMR/MSI mCRC were enrolled. The study flowchart is presented in Figure 1. Patients’ characteristics are reported in Table 1. The median age at diagnosis was 66 years. All patients had a histologically confirmed diagnosis of colorectal carcinoma and they had metastatic disease at the time of the enrollment in the study. Only four of them were metastatic at the time of the diagnosis of the primary CRC. In terms of disease burden at the time of the enrollment, two patients had only one metastatic site, three patients had two sites, seven patients had three sites, and two patients had five different metastatic sites. The most common metastatic sites are reported in Table 1.

**Table 1:** Patients characteristics

Characteristics	Values, *n*/*N*
dMMR/MSI	14/14
Age >65 years	7/14
Male	4/14
Primary tumor location	
Left side	2/14
Right side	8/14
Transverse	1/14
Rectum	1/14
Not available	1/14
Multiple sites	1/14
Metastatic sites at enrollment	
Liver	5/14
Peritoneum	9/14
Lymph nodes	9/14
Lung	9/14
Others	7/14

dMMR, DNA mismatch repair deficiency; MSI, microsatellite instability.

The mutational asset was evaluated: 6 out of 14 patients (42.9%) had a *BRAF*^*V600E*^ mutation, 2 out of 14 (14.3%) had a *RAS* mutation, and the others (6/14) were *BRAF* and *RAS* wild type. One patient had Lynch syndrome, one had *MYH*-associated polyposis syndrome, and a third patient was found to have an *MSH6* variant of uncertain significance.

Pembrolizumab was administered as a first, second, or further line in 35.7%, 43%, and 21% of the cases, respectively.

### Compliance

Three patients never received SABR due to rapid deterioration of their general conditions and death within 2 months after the first course of pembrolizumab. Two of them were *BRAF*^*V600E*^ mutated.

Among the other 11 patients who received SABR, radiotherapy was administered after a median time to the first dose of 21 days, and the second dose of radiotherapy was given 7 days later.

**Figure 2:**
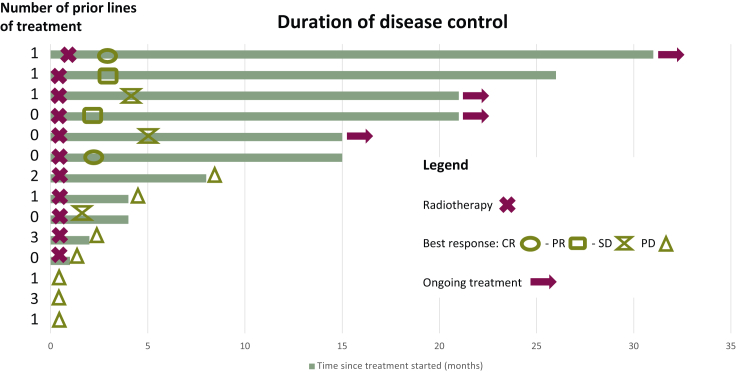
**Duration of disease control.** CR, complete response; PD, progressive disease; PR, partial response; SD, stable disease.

The median duration of treatment was 3.5 months, with six patients maintaining a good response after >15 months (Figure 2).

### Activity

Table 2 shows the overall responses to the treatment: the disease control rate was 50%. Among these, complete response occurred in two patients with a low tumor burden (one and two metastatic sites, respectively). Partial response and stable disease were also observed in patients with a high tumor burden (up to five different sites of disease). The primary resistance (i.e. PD as the best response) among patients who received the combination treatment (4/14) did not correlate with the highest disease burden. Local disease control was achieved in 73% of patients and 50% of them achieved a complete response on the site of SABR.

**Table 2:** Antitumor activity in the intention-to-treat population

Activity	Values, n/N (%)
Overall response (partial response + complete response)	4/14 (28.6)
Complete response	2/14 (14.3)
Partial response	2/14 (14.3)
Stable disease	3/14 (21.4)
Progressive disease	7/14 (50)

Pembrolizumab was allowed to be continued beyond progression, based on clinical judgment. One patient continued pembrolizumab beyond the first progression, but further progression was already observed at 8 months.

#### Toxicity

Any-grade TRAEs occurred in 50% of patients, with G3 events in three patients, although no treatment-related death occurred.

One patient had bowel perforation in the field irradiated by SABR. However, this event could be also related both to disease infiltration of the intestinal wall and to the tumor shrinkage after radiotherapy. Another patient reported, after SABR on metastasis of the right biceps muscle, severe long-lasting neuropathic pain in the right hand. In this case, the tumor mass was very large (11 × 6.6 cm^2^). The ultrasound revealed an irregularity of the musculocutaneous nerve, and, in this specific condition, the infiltration of the tumor could not be excluded as a concomitant cause of chronic pain. However, both patients achieved a complete response with SABR. Finally, two patients who received SABR on lung metastases developed G2 and G3 pneumonia. The patient who reported G3 pneumonia definitely interrupted pembrolizumab. Self-limiting G2 diarrhea occurred in two patients, and these adverse events were probably related to immunotherapy.

## Discussion

At the time this trial was designed, the abscopal effect of immunotherapy combined with radiotherapy was emerging as a promising therapeutic strategy.^22^ Recent research has successfully focused on non-small-cell lung cancer,^17^^,^^18^ melanoma,^19^^,^^23^ and genitourinary cancer.^24^^,^^25^ By contrast, clinical experiences on CRC are extremely scarce, despite some promising results from preclinical studies.^21^^,^^26^ Contrary to the few case reports suggesting that the ICI–radiotherapy combination is safe,^27^, ^28^, ^29^ we are quite concerned about the safety of SABR plus pembrolizumab reported in this study. In fact, 50% of the patients developed TRAEs of any grade and, among these, three patients had G3 toxicity. This level of toxicity is unexpected according to the doses of radiotherapy delivered because this schedule was planned to allow the irradiation of a large tumor volume with a total dose administered lower than in other settings (i.e. oligometastatic). The G3 adverse events were, in particular, pneumonia, bowel perforation, and severe long-lasting neuropathic pain, which significantly impacted the patients’ quality of life. However, a partial role in the toxicities could also be played by the anatomical sites and the tumor bulk that was relevant in each case of G3 toxicity, and that could lighten the impact of SABR on the toxicity profile.

When the trial started in November 2019, the efficacy of pembrolizumab in first-line dMMR/MSI mCRC was not known. Thanks to the trials KEYNOTE-177 and KEYNOTE-164, we now know that anti-PD-1 alone or in combination with anti-cytotoxic T-lymphocyte-associated protein 4 (anti-CTLA-4) given in any line is the most efficacious treatment option in this subset of patients. In addition, data on other checkpoint inhibitors support the notion that ICI given in the first line is more efficacious than when given in later lines.^30^ Our series of patients treated with the combination of radiotherapy and pembrolizumab represents a mixed population where the experimental treatment was given irrespective of the line of treatment: the observed activity level (50% response rate) is in the range of that expected with the checkpoint inhibitors alone under these conditions. Despite the limitation of the small sample size, our study does not give a signal of enhanced pembrolizumab efficacy by SABR. Therefore the postulated potential impact of the immunogenic effect of radiotherapy on systemic control of the disease in the clinic remains controversial.^31^ By contrast, the local control achieved with SABR was extremely interesting (73% of patients obtained a local response), suggesting an important local synergism of immunotherapy and radiotherapy even if at low doses.

There are three potential ways to advance clinical research in this field. The first is conducting larger randomized phase II comparative trials that systematically control the patients’ selection and the modality of radiotherapy delivery.^32^, ^33^, ^34^ The second should privilege the stepwise process of answering which specific volume, timing, and dose of radiation produce the pharmacodynamically relevant release of neoantigens. We believe that in this field, the latter approach is better, although much slower. A third field of expansion of SABR in combination with immunotherapy is its use as maintenance treatment in pMMR/MSS mCRC (NCT03101475 and NCT05375708) instead of ‘hot’ dMMR/MSI tumors that are already highly immune responsive; therefore the addition of radiotherapy could be unnecessary even in the maintenance phase.

In conclusion, these data convey no signal of enhanced systemic efficacy of radiotherapy plus immunotherapy compared with historical data on pembrolizumab alone, even if a synergism in local control cannot be excluded. Further data from larger series could better assess the tolerability of the combination in this setting of patients where immunotherapy alone already affords extraordinary results with excellent toxicity profile.
