# Corrigendum to “Feasibility of stereotactic radiotherapy with pembrolizumab in patients with deficient mismatch repair/microsatellite unstable metastatic colorectal cancer” [ESMO Gastrointestinal Oncology, Volume 5, 100069]

**DOI:** 10.1016/j.esmogo.2025.100279

**Published:** 2026-01-06

**Authors:** A. Gandini, V. Martelli, L. Belgioia, S. Puglisi, M. Cremante, V. Murianni, A. Damassi, C. Pirrone, F. Catalano, M. Grassi, L. Trevisan, S. Vagge, V. Andretta, S. Mammoliti, D. Comandini, G. Fornarini, A. Pessino, A. Pastorino, S. Sciallero, A. Puccini, A.F. Sobrero

**Affiliations:** 1 Department of Internal Medicine and Medical Specialties (Di.M.I.), University of Genoa, Genoa, Italy; 2 Medical Oncology Unit 1, IRCCS Ospedale Policlinico San Martino, Genoa, Italy; 3 Health Science Department (DISSAL), University of Genoa, Genoa, Italy; 4 Radiation Oncology Department, IRCCS Ospedale Policlinico San Martino, Genoa, Italy; 5 Unit of Hereditary Cancer, IRCCS Ospedale Policlinico San Martino, Genoa, Italy; 6 Radiotherapy Department, E.O. Ospedali Galliera, Genoa, Italy

The authors regret that in the original publication it was not clear that this study was conducted as a compassionate use clinical trial. In the “Study design and treatment” subheading of the Patients and Methods section, the correct statement should read as follows:

This monocentric interventional prospective compassionate use study was conducted at IRCCS Ospedale Policlinico San Martino in Genoa, Italy.

The authors would like to apologise for any inconvenience caused.

